# Tetra­kis[μ-2-(meth­oxy­carbon­yl)benzoato-κ^2^ *O* ^1^:*O* ^1′^]bis­[(acetonitrile-κ*N*)copper(II)](*Cu*—*Cu*)

**DOI:** 10.1107/S1600536812049410

**Published:** 2012-12-08

**Authors:** Jing-lin Wang, Cai-rong Wang, Zhi-jun Wang, Bin-sheng Yang

**Affiliations:** a Department of Chemistry, Changzhi University, Changzhi, Shanxi 046011, People’s Republic of China; b Institute of Molecular Science, Shanxi University, Taiyuan, Shanxi 030006, People’s Republic of China

## Abstract

In the binuclear copper(II) title complex, [Cu_2_(C_9_H_7_O_4_)_4_(C_2_H_3_N)_2_], an inversion centre is situtated at the mid-point of the Cu—Cu bond. The Cu^II^ atom together with its four coordinated O atoms are in a distorted planar square arrangement while the nitro­gen and the other Cu^II^ atom are located in apical positions. The whole mol­ecule looks like a paddle-wheel. In the crystal, chains are assembled along the *b* axis through C—H⋯O hydrogen bonds and slipped π–π inter­actions between the benzene rings of neighbouring mol­ecules [centroid–centroid distance = 3.6929 (3) Å and slippage = 0.641 (1) Å].

## Related literature

For a review on related binuclear Cu^II^ carboxyl­ato compounds with subnormal magnetic moments, see: Kato *et al.* (1964 ▶). For the electrochemical behavior of related compounds, see: Reinhard *et al.* (2003 ▶). For the synthesis of related compounds, see: Liu *et al.* (2008 ▶).
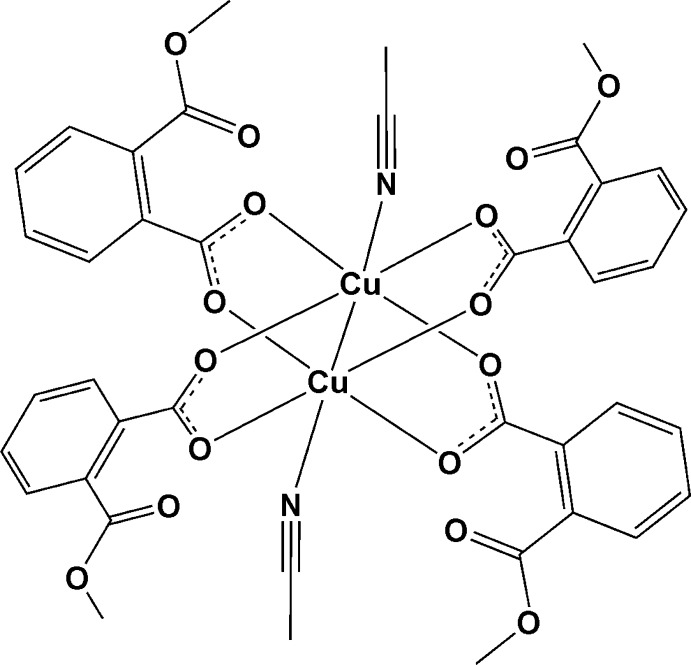


## Experimental

### 

#### Crystal data

[Cu_2_(C_9_H_7_O_4_)_4_(C_2_H_3_N)_2_]
*M* *_r_* = 925.77
Triclinic, 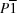

*a* = 8.2332 (10) Å
*b* = 10.5730 (13) Å
*c* = 12.6673 (15) Å
α = 104.774 (1)°
β = 108.061 (2)°
γ = 91.152 (1)°
*V* = 1007.8 (2) Å^3^
*Z* = 1
Mo *K*α radiation
μ = 1.13 mm^−1^
*T* = 298 K
0.41 × 0.30 × 0.27 mm

#### Data collection

Bruker SMART CCD area-detector diffractometer
Absorption correction: multi-scan (*SADABS*; Bruker, 2001 ▶) *T*_min_ = 0.654, *T*_max_ = 0.750
5143 measured reflections
3466 independent reflections
2906 reflections with *I* > 2σ(*I*)
*R*_int_ = 0.022

#### Refinement

*R*[*F* ^2^ > 2σ(*F* ^2^)] = 0.037
*wR*(*F* ^2^) = 0.105
*S* = 1.07
3466 reflections
271 parameters
H-atom parameters constrained
Δρ_max_ = 0.55 e Å^−3^
Δρ_min_ = −0.32 e Å^−3^

### 

Data collection: *SMART* (Bruker, 1999 ▶); cell refinement: *SAINT* (Bruker, 1999 ▶); data reduction: *SAINT*; program(s) used to solve structure: *SHELXS97* (Sheldrick, 2008 ▶); program(s) used to refine structure: *SHELXL97* (Sheldrick, 2008 ▶); molecular graphics: *SHELXTL* (Sheldrick, 2008 ▶); software used to prepare material for publication: *SHELXTL*.

## Supplementary Material

Crystal structure: contains datablock(s) I, global. DOI: 10.1107/S1600536812049410/lr2089sup1.cif

Structure factors: contains datablock(s) I. DOI: 10.1107/S1600536812049410/lr2089Isup2.hkl

Additional supplementary materials:  crystallographic information; 3D view; checkCIF report

## Figures and Tables

**Table 1:** Hydrogen-bond geometry (Å, °)

*D*—H⋯*A*	*D*—H	H⋯*A*	*D*⋯*A*	*D*—H⋯*A*
C5—H5⋯O5^i^	0.93	2.51	3.379 (4)	156

Symmetry code: (i) 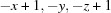.

**Fig. 1.**
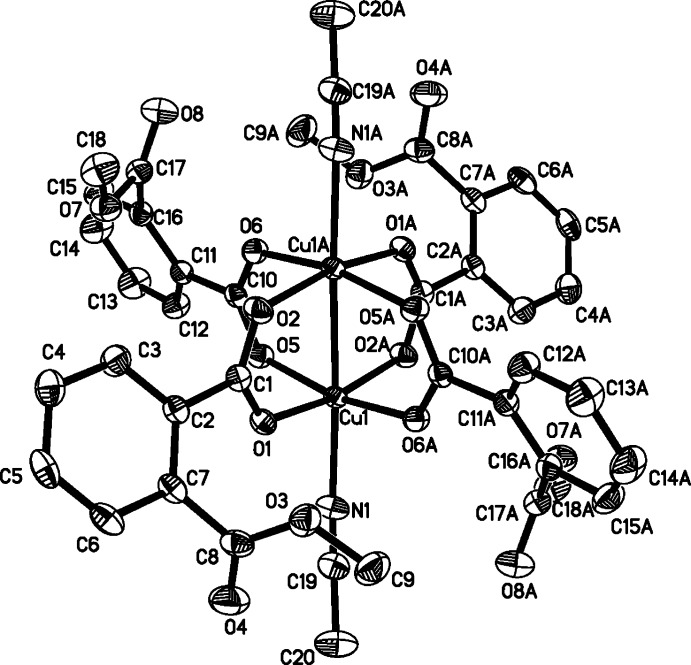
Molecular structure of the title compound with displacement ellipsoids drawn at the 30% probability level. The H atoms attached to C atoms were omitted for clarity. Atoms with the A label are generated by the (1 - x, 1 - y, 1 - z) symmetry operation.

**Fig. 2.**
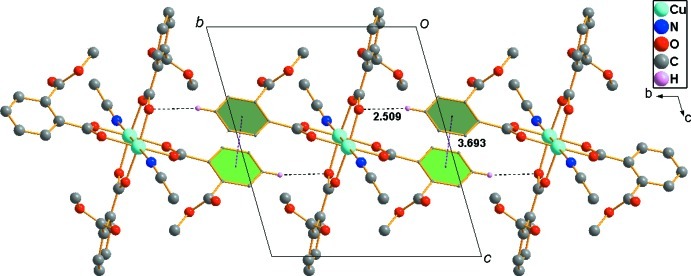
View of the crystal packing along the a axis. Hydrogen-bonding and π-π interactions are represented by black dashed lines and pink dashed lines, respectively (all distances in Å). For the sake of clarity, H atoms not involved in hydrogen bonding have been omitted.

## supplementary crystallographic information

### Comment

A large number of binuclear Cu^II^ carboxylato compounds are an attractive target of chemical research due to their magnetism (Kato *et al.*, 1964) and electrochemical behavior (Reinhard *et al.*, 2003). In general, binuclear copper (II) carboxylates consist of four three-atom bridges, uniting two contiguous copper(II) ions and exhibit a paddle-wheel cage structure.

Herein we report synthesis and crystal structure of binuclear copper(II) carboxylato compound with 2-(methoxycarbonyl)benzoic acid acting as a bidentate chelating ligand. Each copper(II) is coordinated by four carboxylate O donor atoms from four ligands, and by N donor atoms from the solvent molecule, The Cu—O distances and related angles are all within expected ranges (Kato *et al.*, 1964) and Cu—N distance is 2.186 (3) Å. A binuclear copper carboxylate unit is formed by four (HL) ligands and two Cu centres with a Cu—Cu separation of 2.6662 (7) Å.

In the crystal structure, weak C—H···O hydrogen bonds (H···O distance of 2.5087 (22) Å) and π-π interactions (centroid–centroid distance of 3.6929 (3) Å) link the molecules into an infinite one-dimensional chain extending along the *b* axis.

### Experimental

The title complex was prepared by adapting a reported procedure (Liu *et al.*, 2008) by stirring a methanolic solutions of 2-(methoxycarbonyl)benzoic acid (180.0 mg, 1.0 mmol) and NaOH (40.0 mg, 1.0 mmol) for 30 min at room temperature. Then, 10 ml of a methanol solution containing Cu(NO_3_)_2_.3H_2_O (121 mg, 0.5 mmol) was added to the mixture, the blue precipitate obtained was separated by filtration, washed with methanol and dried. The blue powder was dissolved in acetonitrile, and single crystals of the title complex suitable for X-ray analysis were obtained after slow evaporation at room temperature for several weeks.

### Refinement

H atoms attached to C atoms are placed in geometrically idealized position, with C–H=0.93 and 0.96 Å, for CH and CH_3_ groups, respectively, and with *U*_iso_(H)=1.2*U*_eq_ (C*_sp_*_2_) or 1.5*U*_eq_ (C*_sp_*_3_).

### Crystal data

**Table:**

[Cu_2_(C_9_H_7_O_4_)_4_(C_2_H_3_N)_2_]	*Z* = 1
*M**_r_* = 925.77	*F*(000) = 474
Triclinic, *P*1	*D*_x_ = 1.525 Mg m^−^^3^
Hall symbol: -P 1	Mo *K*α radiation, λ = 0.71073 Å
*a* = 8.2332 (10) Å	Cell parameters from 2861 reflections
*b* = 10.5730 (13) Å	θ = 2.3–27.6°
*c* = 12.6673 (15) Å	µ = 1.13 mm^−^^1^
α = 104.774 (1)°	*T* = 298 K
β = 108.061 (2)°	Block, blue
γ = 91.152 (1)°	0.41 × 0.30 × 0.27 mm
*V* = 1007.8 (2) Å^3^	

### Data collection

**Table:**

Bruker SMART CCD area-detector diffractometer	3466 independent reflections
Radiation source: fine-focus sealed tube	2906 reflections with *I* > 2σ(*I*)
Graphite monochromator	*R*_int_ = 0.022
phi and ω scans	θ_max_ = 25.0°, θ_min_ = 1.8°
Absorption correction: multi-scan (*SADABS*; Bruker, 2001)	*h* = −9→9
*T*_min_ = 0.654, *T*_max_ = 0.750	*k* = −12→12
5143 measured reflections	*l* = −11→15

### Refinement

**Table:**

Refinement on *F*^2^	Primary atom site location: structure-invariant direct methods
Least-squares matrix: full	Secondary atom site location: difference Fourier map
*R*[*F*^2^ > 2σ(*F*^2^)] = 0.037	Hydrogen site location: inferred from neighbouring sites
*wR*(*F*^2^) = 0.105	H-atom parameters constrained
*S* = 1.07	*w* = 1/[σ^2^(*F*_o_^2^) + (0.0576*P*)^2^ + 0.2799*P*] where *P* = (*F*_o_^2^ + 2*F*_c_^2^)/3
3466 reflections	(Δ/σ)_max_ = 0.001
271 parameters	Δρ_max_ = 0.55 e Å^−^^3^
0 restraints	Δρ_min_ = −0.32 e Å^−^^3^

### Special details

**Table:**

Geometry. All e.s.d.'s (except the e.s.d. in the dihedral angle between two l.s. planes) are estimated using the full covariance matrix. The cell e.s.d.'s are taken into account individually in the estimation of e.s.d.'s in distances, angles and torsion angles; correlations between e.s.d.'s in cell parameters are only used when they are defined by crystal symmetry. An approximate (isotropic) treatment of cell e.s.d.'s is used for estimating e.s.d.'s involving l.s. planes.
Refinement. Refinement of *F*^2^ against ALL reflections. The weighted *R*-factor *wR* and goodness of fit *S* are based on *F*^2^, conventional *R*-factors *R* are based on *F*, with *F* set to zero for negative *F*^2^. The threshold expression of *F*^2^ > σ(*F*^2^) is used only for calculating *R*-factors(gt) *etc*. and is not relevant to the choice of reflections for refinement. *R*-factors based on *F*^2^ are statistically about twice as large as those based on *F*, and *R*- factors based on ALL data will be even larger.

### Fractional atomic coordinates and isotropic or equivalent isotropic displacement parameters (Å^2^)

**Table:**

	*x*	*y*	*z*	*U*_iso_*/*U*_eq_	
Cu1	0.66718 (4)	0.48669 (3)	0.52396 (3)	0.02864 (14)	
N1	0.9334 (4)	0.4460 (3)	0.5907 (3)	0.0511 (7)	
O1	0.5939 (3)	0.33112 (19)	0.56430 (17)	0.0356 (5)	
O2	0.3117 (3)	0.3521 (2)	0.52217 (19)	0.0407 (5)	
O3	0.6624 (3)	0.3323 (2)	0.8074 (2)	0.0519 (6)	
O4	0.8468 (4)	0.1920 (3)	0.7599 (3)	0.0758 (9)	
O5	0.6016 (3)	0.3852 (2)	0.36022 (17)	0.0370 (5)	
O6	0.3193 (3)	0.4033 (2)	0.32214 (17)	0.0373 (5)	
O7	0.1070 (3)	0.1505 (2)	0.2080 (2)	0.0489 (6)	
O8	−0.0269 (3)	0.2908 (3)	0.1135 (2)	0.0591 (7)	
C1	0.4421 (4)	0.2953 (3)	0.5562 (2)	0.0320 (7)	
C2	0.4171 (4)	0.1705 (3)	0.5883 (3)	0.0341 (7)	
C3	0.2681 (4)	0.0870 (3)	0.5272 (3)	0.0481 (9)	
H3	0.1831	0.1115	0.4703	0.058*	
C4	0.2426 (5)	−0.0317 (3)	0.5485 (3)	0.0531 (9)	
H4	0.1406	−0.0861	0.5072	0.064*	
C5	0.3681 (5)	−0.0695 (3)	0.6311 (3)	0.0497 (9)	
H5	0.3519	−0.1502	0.6451	0.060*	
C6	0.5178 (5)	0.0117 (3)	0.6930 (3)	0.0475 (8)	
H6	0.6026	−0.0142	0.7490	0.057*	
C7	0.5432 (4)	0.1324 (3)	0.6725 (3)	0.0366 (7)	
C8	0.7035 (5)	0.2203 (4)	0.7487 (3)	0.0466 (8)	
C9	0.8041 (6)	0.4269 (4)	0.8868 (3)	0.0730 (13)	
H9A	0.8914	0.3821	0.9281	0.109*	
H9B	0.7641	0.4889	0.9405	0.109*	
H9C	0.8510	0.4726	0.8446	0.109*	
C10	0.4473 (4)	0.3629 (3)	0.2946 (2)	0.0314 (7)	
C11	0.4130 (4)	0.2844 (3)	0.1715 (2)	0.0340 (7)	
C12	0.5451 (4)	0.2722 (3)	0.1247 (3)	0.0458 (8)	
H12	0.6562	0.3065	0.1721	0.055*	
C13	0.5161 (5)	0.2104 (4)	0.0094 (3)	0.0612 (10)	
H13	0.6066	0.2032	−0.0202	0.073*	
C14	0.3510 (5)	0.1593 (4)	−0.0613 (3)	0.0667 (11)	
H14	0.3299	0.1182	−0.1392	0.080*	
C15	0.2173 (5)	0.1693 (4)	−0.0162 (3)	0.0549 (10)	
H15	0.1066	0.1344	−0.0643	0.066*	
C16	0.2458 (4)	0.2306 (3)	0.0994 (3)	0.0380 (7)	
C17	0.0956 (4)	0.2320 (3)	0.1417 (3)	0.0413 (8)	
C18	−0.0319 (5)	0.1442 (4)	0.2536 (3)	0.0629 (11)	
H18A	−0.0247	0.2246	0.3119	0.094*	
H18B	−0.0240	0.0716	0.2869	0.094*	
H18C	−0.1396	0.1323	0.1925	0.094*	
C19	1.0408 (4)	0.4208 (4)	0.6578 (3)	0.0467 (8)	
C20	1.1799 (5)	0.3891 (5)	0.7485 (4)	0.0714 (12)	
H20A	1.1928	0.4511	0.8214	0.107*	
H20B	1.2852	0.3933	0.7313	0.107*	
H20C	1.1531	0.3019	0.7524	0.107*	

### Atomic displacement parameters (Å^2^)

**Table:**

	*U*^11^	*U*^22^	*U*^33^	*U*^12^	*U*^13^	*U*^23^
Cu1	0.0319 (2)	0.0312 (2)	0.0265 (2)	0.00672 (14)	0.01079 (15)	0.01251 (15)
N1	0.0412 (16)	0.062 (2)	0.0581 (19)	0.0198 (14)	0.0169 (14)	0.0284 (16)
O1	0.0416 (12)	0.0331 (11)	0.0384 (12)	0.0063 (9)	0.0153 (9)	0.0181 (9)
O2	0.0420 (12)	0.0384 (12)	0.0496 (13)	0.0073 (10)	0.0158 (10)	0.0245 (10)
O3	0.0625 (15)	0.0520 (15)	0.0372 (13)	0.0001 (12)	0.0093 (11)	0.0144 (11)
O4	0.0478 (16)	0.082 (2)	0.093 (2)	0.0159 (15)	0.0066 (15)	0.0341 (18)
O5	0.0397 (12)	0.0452 (13)	0.0268 (11)	0.0078 (10)	0.0105 (9)	0.0111 (9)
O6	0.0379 (12)	0.0428 (13)	0.0302 (11)	0.0077 (10)	0.0123 (9)	0.0067 (9)
O7	0.0455 (13)	0.0544 (15)	0.0515 (14)	0.0058 (11)	0.0150 (11)	0.0236 (12)
O8	0.0462 (14)	0.0785 (19)	0.0617 (17)	0.0205 (13)	0.0176 (12)	0.0339 (14)
C1	0.0418 (18)	0.0336 (16)	0.0242 (14)	0.0047 (14)	0.0140 (13)	0.0099 (12)
C2	0.0412 (17)	0.0319 (16)	0.0350 (16)	0.0061 (13)	0.0179 (13)	0.0123 (13)
C3	0.050 (2)	0.045 (2)	0.048 (2)	−0.0028 (16)	0.0097 (16)	0.0195 (16)
C4	0.059 (2)	0.042 (2)	0.061 (2)	−0.0027 (17)	0.0232 (18)	0.0148 (17)
C5	0.071 (2)	0.0314 (18)	0.065 (2)	0.0131 (17)	0.041 (2)	0.0215 (17)
C6	0.061 (2)	0.045 (2)	0.054 (2)	0.0211 (17)	0.0298 (18)	0.0284 (17)
C7	0.0462 (18)	0.0360 (17)	0.0393 (17)	0.0120 (14)	0.0237 (14)	0.0176 (14)
C8	0.049 (2)	0.051 (2)	0.048 (2)	0.0102 (17)	0.0129 (16)	0.0299 (17)
C9	0.084 (3)	0.063 (3)	0.051 (2)	−0.009 (2)	−0.011 (2)	0.020 (2)
C10	0.0415 (17)	0.0281 (15)	0.0292 (15)	0.0069 (13)	0.0129 (14)	0.0142 (12)
C11	0.0412 (17)	0.0329 (16)	0.0287 (15)	0.0074 (13)	0.0118 (13)	0.0089 (12)
C12	0.0457 (19)	0.055 (2)	0.0348 (18)	0.0047 (16)	0.0144 (15)	0.0086 (15)
C13	0.063 (2)	0.081 (3)	0.042 (2)	0.010 (2)	0.0296 (19)	0.0068 (19)
C14	0.072 (3)	0.086 (3)	0.0330 (19)	0.011 (2)	0.0174 (19)	−0.0013 (19)
C15	0.051 (2)	0.065 (2)	0.0349 (19)	0.0045 (18)	0.0053 (16)	0.0020 (17)
C16	0.0424 (17)	0.0359 (17)	0.0335 (17)	0.0065 (14)	0.0110 (14)	0.0076 (13)
C17	0.0405 (18)	0.0426 (19)	0.0361 (17)	0.0053 (15)	0.0070 (14)	0.0094 (14)
C18	0.054 (2)	0.081 (3)	0.061 (3)	−0.002 (2)	0.0207 (19)	0.030 (2)
C19	0.0412 (19)	0.057 (2)	0.053 (2)	0.0121 (16)	0.0240 (17)	0.0224 (18)
C20	0.059 (2)	0.107 (4)	0.061 (3)	0.027 (2)	0.018 (2)	0.045 (2)

### Geometric parameters (Å, º)

**Table:**

Cu1—O2^i^	1.959 (2)	C5—H5	0.9300
Cu1—O6^i^	1.967 (2)	C6—C7	1.390 (4)
Cu1—O5	1.973 (2)	C6—H6	0.9300
Cu1—O1	1.9775 (19)	C7—C8	1.497 (5)
Cu1—N1	2.186 (3)	C9—H9A	0.9600
Cu1—Cu1^i^	2.6662 (7)	C9—H9B	0.9600
N1—C19	1.111 (4)	C9—H9C	0.9600
O1—C1	1.264 (4)	C10—C11	1.503 (4)
O2—C1	1.253 (4)	C11—C12	1.385 (5)
O2—Cu1^i^	1.9590 (19)	C11—C16	1.405 (4)
O3—C8	1.338 (4)	C12—C13	1.380 (5)
O3—C9	1.445 (4)	C12—H12	0.9300
O4—C8	1.197 (4)	C13—C14	1.382 (6)
O5—C10	1.263 (3)	C13—H13	0.9300
O6—C10	1.259 (4)	C14—C15	1.383 (5)
O6—Cu1^i^	1.967 (2)	C14—H14	0.9300
O7—C17	1.335 (4)	C15—C16	1.385 (4)
O7—C18	1.439 (4)	C15—H15	0.9300
O8—C17	1.202 (4)	C16—C17	1.492 (5)
C1—C2	1.505 (4)	C18—H18A	0.9600
C2—C3	1.380 (4)	C18—H18B	0.9600
C2—C7	1.388 (4)	C18—H18C	0.9600
C3—C4	1.375 (5)	C19—C20	1.464 (5)
C3—H3	0.9300	C20—H20A	0.9600
C4—C5	1.371 (5)	C20—H20B	0.9600
C4—H4	0.9300	C20—H20C	0.9600
C5—C6	1.373 (5)		
			
O2^i^—Cu1—O6^i^	88.57 (9)	O3—C8—C7	109.7 (3)
O2^i^—Cu1—O5	89.00 (9)	O3—C9—H9A	109.5
O6^i^—Cu1—O5	167.59 (8)	O3—C9—H9B	109.5
O2^i^—Cu1—O1	167.74 (9)	H9A—C9—H9B	109.5
O6^i^—Cu1—O1	89.31 (9)	O3—C9—H9C	109.5
O5—Cu1—O1	90.49 (9)	H9A—C9—H9C	109.5
O2^i^—Cu1—N1	103.49 (10)	H9B—C9—H9C	109.5
O6^i^—Cu1—N1	90.66 (10)	O6—C10—O5	125.9 (3)
O5—Cu1—N1	101.74 (10)	O6—C10—C11	116.7 (2)
O1—Cu1—N1	88.60 (10)	O5—C10—C11	117.3 (3)
O2^i^—Cu1—Cu1^i^	86.83 (6)	C12—C11—C16	118.7 (3)
O6^i^—Cu1—Cu1^i^	83.15 (6)	C12—C11—C10	120.0 (3)
O5—Cu1—Cu1^i^	84.57 (6)	C16—C11—C10	121.2 (3)
O1—Cu1—Cu1^i^	80.93 (6)	C13—C12—C11	121.7 (3)
N1—Cu1—Cu1^i^	167.87 (8)	C13—C12—H12	119.1
C19—N1—Cu1	151.4 (3)	C11—C12—H12	119.1
C1—O1—Cu1	125.83 (19)	C12—C13—C14	119.3 (4)
C1—O2—Cu1^i^	120.03 (19)	C12—C13—H13	120.3
C8—O3—C9	116.4 (3)	C14—C13—H13	120.3
C10—O5—Cu1	122.1 (2)	C13—C14—C15	120.0 (3)
C10—O6—Cu1^i^	124.20 (19)	C13—C14—H14	120.0
C17—O7—C18	115.9 (3)	C15—C14—H14	120.0
O2—C1—O1	126.4 (3)	C14—C15—C16	120.9 (3)
O2—C1—C2	117.5 (3)	C14—C15—H15	119.5
O1—C1—C2	116.1 (3)	C16—C15—H15	119.5
C3—C2—C7	118.7 (3)	C15—C16—C11	119.4 (3)
C3—C2—C1	118.9 (3)	C15—C16—C17	117.3 (3)
C7—C2—C1	122.3 (3)	C11—C16—C17	123.3 (3)
C4—C3—C2	121.3 (3)	O8—C17—O7	123.6 (3)
C4—C3—H3	119.3	O8—C17—C16	124.9 (3)
C2—C3—H3	119.3	O7—C17—C16	111.3 (3)
C5—C4—C3	119.7 (3)	O7—C18—H18A	109.5
C5—C4—H4	120.1	O7—C18—H18B	109.5
C3—C4—H4	120.1	H18A—C18—H18B	109.5
C4—C5—C6	120.1 (3)	O7—C18—H18C	109.5
C4—C5—H5	120.0	H18A—C18—H18C	109.5
C6—C5—H5	120.0	H18B—C18—H18C	109.5
C5—C6—C7	120.3 (3)	N1—C19—C20	178.4 (4)
C5—C6—H6	119.8	C19—C20—H20A	109.5
C7—C6—H6	119.8	C19—C20—H20B	109.5
C2—C7—C6	119.8 (3)	H20A—C20—H20B	109.5
C2—C7—C8	122.5 (3)	C19—C20—H20C	109.5
C6—C7—C8	117.6 (3)	H20A—C20—H20C	109.5
O4—C8—O3	125.1 (4)	H20B—C20—H20C	109.5
O4—C8—C7	125.1 (4)		
			
O2^i^—Cu1—N1—C19	131.8 (6)	C5—C6—C7—C8	175.7 (3)
O6^i^—Cu1—N1—C19	43.1 (7)	C9—O3—C8—O4	2.5 (5)
O5—Cu1—N1—C19	−136.4 (7)	C9—O3—C8—C7	178.8 (3)
O1—Cu1—N1—C19	−46.2 (7)	C2—C7—C8—O4	−125.1 (4)
Cu1^i^—Cu1—N1—C19	−15.9 (10)	C6—C7—C8—O4	58.7 (5)
O2^i^—Cu1—O1—C1	4.0 (5)	C2—C7—C8—O3	58.5 (4)
O6^i^—Cu1—O1—C1	84.0 (2)	C6—C7—C8—O3	−117.7 (3)
O5—Cu1—O1—C1	−83.6 (2)	Cu1^i^—O6—C10—O5	0.6 (4)
N1—Cu1—O1—C1	174.7 (2)	Cu1^i^—O6—C10—C11	−177.57 (17)
Cu1^i^—Cu1—O1—C1	0.8 (2)	Cu1—O5—C10—O6	1.9 (4)
O2^i^—Cu1—O5—C10	−89.3 (2)	Cu1—O5—C10—C11	−179.96 (17)
O6^i^—Cu1—O5—C10	−10.6 (5)	O6—C10—C11—C12	159.6 (3)
O1—Cu1—O5—C10	78.5 (2)	O5—C10—C11—C12	−18.8 (4)
N1—Cu1—O5—C10	167.1 (2)	O6—C10—C11—C16	−15.6 (4)
Cu1^i^—Cu1—O5—C10	−2.4 (2)	O5—C10—C11—C16	166.0 (3)
Cu1^i^—O2—C1—O1	−0.4 (4)	C16—C11—C12—C13	0.8 (5)
Cu1^i^—O2—C1—C2	−178.87 (18)	C10—C11—C12—C13	−174.5 (3)
Cu1—O1—C1—O2	−0.5 (4)	C11—C12—C13—C14	0.0 (6)
Cu1—O1—C1—C2	177.95 (18)	C12—C13—C14—C15	−0.6 (7)
O2—C1—C2—C3	34.7 (4)	C13—C14—C15—C16	0.3 (6)
O1—C1—C2—C3	−143.9 (3)	C14—C15—C16—C11	0.6 (5)
O2—C1—C2—C7	−149.3 (3)	C14—C15—C16—C17	−177.5 (4)
O1—C1—C2—C7	32.1 (4)	C12—C11—C16—C15	−1.1 (5)
C7—C2—C3—C4	0.3 (5)	C10—C11—C16—C15	174.1 (3)
C1—C2—C3—C4	176.4 (3)	C12—C11—C16—C17	176.8 (3)
C2—C3—C4—C5	−0.9 (6)	C10—C11—C16—C17	−7.9 (4)
C3—C4—C5—C6	0.8 (6)	C18—O7—C17—O8	−5.2 (5)
C4—C5—C6—C7	−0.1 (5)	C18—O7—C17—C16	180.0 (3)
C3—C2—C7—C6	0.4 (5)	C15—C16—C17—O8	−66.9 (5)
C1—C2—C7—C6	−175.5 (3)	C11—C16—C17—O8	115.1 (4)
C3—C2—C7—C8	−175.7 (3)	C15—C16—C17—O7	107.8 (3)
C1—C2—C7—C8	8.4 (5)	C11—C16—C17—O7	−70.2 (4)
C5—C6—C7—C2	−0.5 (5)	Cu1—N1—C19—C20	−23 (16)

Symmetry code: (i) −*x*+1, −*y*+1, −*z*+1.

### Hydrogen-bond geometry (Å, º)

**Table:**

*D*—H···*A*	*D*—H	H···*A*	*D*···*A*	*D*—H···*A*
C5—H5···O5^ii^	0.93	2.51	3.379 (4)	156

Symmetry code: (ii) −*x*+1, −*y*, −*z*+1.
